# Integrin-Dependent Cell–Matrix Adhesion in Endothelial Health and Disease

**DOI:** 10.1161/CIRCRESAHA.122.322332

**Published:** 2023-02-03

**Authors:** Jurjan Aman, Coert Margadant

**Affiliations:** Department of Pulmonology, Amsterdam University Medical Center, the Netherlands (J.A.).; Department of Medical Oncology, Amsterdam University Medical Center, the NetherlandsInstitute of Biology, Leiden University, the Netherlands (C.M.).

**Keywords:** angiogenesis, cell-matrix adhesions, edema, endothelium, inflammation, integrins

## Abstract

The endothelium is a dynamic, semipermeable layer lining all blood vessels, regulating blood vessel formation and barrier function. Proper composition and function of the endothelial barrier are required for fluid homeostasis, and clinical conditions characterized by barrier disruption are associated with severe morbidity and high mortality rates. Endothelial barrier properties are regulated by cell–cell junctions and intracellular signaling pathways governing the cytoskeleton, but recent insights indicate an increasingly important role for integrin-mediated cell–matrix adhesion and signaling in endothelial barrier regulation. Here, we discuss diseases characterized by endothelial barrier disruption, and provide an overview of the composition of endothelial cell–matrix adhesion complexes and associated signaling pathways, their crosstalk with cell–cell junctions, and with other receptors. We further present recent insights into the role of cell–matrix adhesions in the developing and mature/adult endothelium of various vascular beds, and discuss how the dynamic regulation and turnover of cell–matrix adhesions regulates endothelial barrier function in (patho)physiological conditions like angiogenesis, inflammation and in response to hemodynamic stress. Finally, as clinical conditions associated with vascular leak still lack direct treatment, we focus on how understanding of endothelial cell–matrix adhesion may provide novel targets for treatment, and discuss current translational challenges and future perspectives.

The vascular endothelium forms a emipermeable barrier that regulates the traffic of leukocytes, fluids, and proteins. Barrier regulation is essential to facilitate physiological transport of fluid and nutrients, while preventing excessive vascular leakage. Endothelial hyperpermeability can cause tissue damage or life-threatening edema, associated with a variety of pathological conditions, including coronavirus disease (COVID-19), sepsis, acute respiratory distress syndrome (ARDS), angioedema and visceral edema, stroke, retinopathies, and capillary leak syndrome (Figure 1). Although mentioned conditions carry high mortality (eg, 35% of ARDS patients dies within 28 days after diagnosis), there is no direct treatment to reverse vascular leakage.

**Figure 1.**
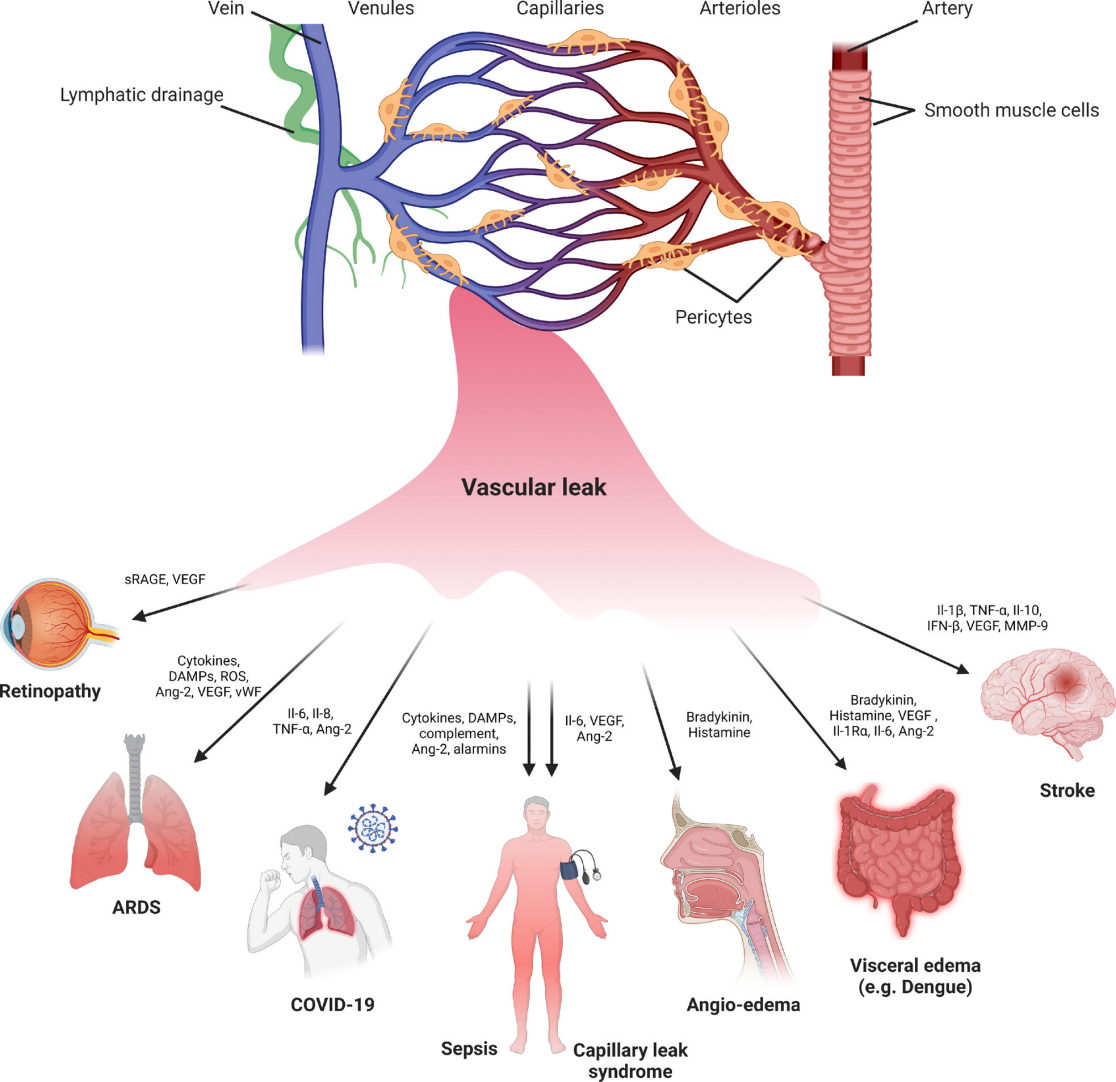
**Diseases associated with clinical vascular leak.** Depicted is the vascular tree and associated cell types. Clinically relevant vascular leak predominantly takes place at the capillary and venular level. The clinical sequelae of vascular leak may vary according to etiological factors involved and to the affected vascular bed, ranging from general to organ-specific manifestations like in ARDS and angioedema. In addition, the vasoactive substances involved in vascular leak may vary according to clinical condition. Of note, many of these cytokines have been identified in cross-sectional studies. Ang-2 sensitizes the endothelium to vasoactive agents, among others via integrin binding. ARDS indicates acute respiratory distress syndrome; Ang-2, angiopoietin-2; DAMPs, disease-associated molecular patterns; IFN, interferon; Il, interleukin; Il1R, interleukin 1 receptor; MMP, matrix metalloproteinase; ROS, reactive oxygen species; sRAGE, soluble receptor for advanced glycation end products; TNF, tumor necrosis factor; VEGF, vascular endothelial growth factor; and vWF, von Willebrand Factor. Created with Biorender.com.

Large blood vessels generally contain a single layer of endothelial cells (ECs) and associated pericytes adhering to an underlying basement membrane (BM), surrounded by layers of vascular smooth muscle cells and a sheath of connective tissue. Toward the periphery of the vascular network, less vascular smooth muscle cells are observed in smaller vessels like arterioles and venules, whereas capillaries are solely built by ECs and a BM, together with supporting pericytes. The endothelium maintains barrier function through cell–cell adhesion complexes, including tight junctions and adherens junctions (AJs; Figure 2A). AJs have received most attention as regulators of dynamic cell–cell interactions during processes such as angiogenesis or barrier disruption and recovery. AJs are assembled by homotypic interactions of vascular endothelial-cadherin (VE-cadherin), a transmembrane receptor of the cadherin family that connects intracellularly to actin filaments via adapter proteins including the catenins^1^ (Figure 2A and 2B). In addition, ECs tightly adhere to the extracellular matrix (ECM; being the BM in mature vessels and the interstitial matrix in angiogenic vessels) via heterodimeric transmembrane receptors called integrins, which, like VE-cadherin, connect to the actin cytoskeleton through a variety of adapters. Several integrin-based adhesion complexes have been identified, including nascent adhesions, focal adhesions (FAs), and fibrillar adhesions (Figure 2C and 2D), the formation of which depends on tightly balanced activities of the small GTPases Rho (Ras homologous) and Rac (Ras-related C3 botulinum toxin substrate).^2^ FAs can contain up to hundreds of different proteins, and do not only stimulate cell adhesion and migration, but are also major signaling centers regulating cell survival, proliferation, and gene expression.

**Figure 2.**
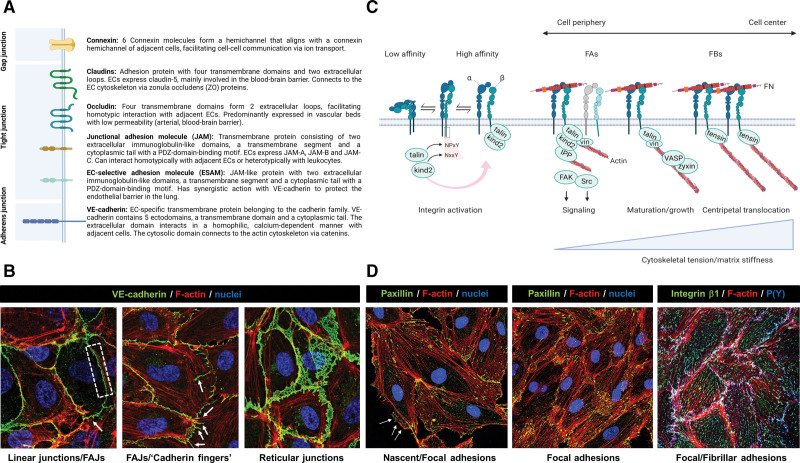
**Basics of endothelial cell (EC)–cell and cell–extracellular matrix (ECM) adhesion. A**, Properties of cell–cell junction proteins in ECs. Created with Biorender.com. **B**, Examples of vascular endothelial-cadherin (VE-cadherin) distribution in AJs in endothelial cells. The dotted box in the **left** image indicates linear junctions, arrows point to focal AJs (FAJs). In the **middle** image, arrows indicate cadherin fingers. **C**, Integrins are αβ heterodimeric cell-surface receptors that exist in a dynamic equilibrium between the bent conformation (with low affinity for ligand) and the extended conformation (with high affinity for ligand). The shift from bent-to-extended is called integrin activation and is promoted by binding of talin (talin-1 or talin-2) and kindlin (kindlin-1, -2, or -3) to the NPxY/NxxY motifs in the β-cytoplasmic tail, which can also recruit a variety of other proteins such as tensin. Ligand-bound integrins are connected to actin filaments and recruit many other factors with a structural, adapter, or signaling role, thus building adhesion complexes such as nascent adhesions. These can contain several different integrins, are not connected to actin stress fibers, and are promoted by Rac activity. In contrast, FAs are connected to stress fibers, and as many FA components are recruited in a tension-dependent manner, they grow and mature after increased RhoA activity. Whereas FAs are predominantly peripheral, specialized adhesions called FBs arise from the translocation of integrin α5β1 in a RhoA- and tensin-dependent manner from the cell periphery to the cell center, a process associated with cell contractility and FN fibrillogenesis. Created with Biorender.com. **D**, Examples of cell-ECM adhesions in ECs. Paxillin and P(Y) mark nascent adhesions (arrows)/FAs, elongated central adhesions positive for β1 are FBs. FAs indicates focal adhesions; FAK, focal adhesion kinase; FBs, fibrillar adhesions; FN, fibronectin; IPP, ILK-PINCH-parvin complex; kind2, kindlin-2; FN, fibronectin; p(Y), phosphorylated tyrosine; Src, proto-oncogene tyrosine-protein kinase; VASP, vasodilator-stimulated phosphoprotein; and vin, vinculin.

Both cell–cell and cell–matrix adhesions are highly dynamic structures, and extensive remodeling occurs in response to changes in blood flow, as well as permeability-inducing agents released upon infection, inflammation, or wounding, including vascular endothelial growth factor (VEGF), thrombin, histamine, interleukin 1β, interleukin 6, and tumor necrosis factor α (TNF-α).^3^ In addition, it becomes increasingly apparent that cell–matrix adhesions and cell–cell junctions share a variety of components, and that there is extensive crosstalk between the 2 systems.

The importance of cell–cell adhesions and the role of actomyosin dynamics in endothelial barrier formation and maintenance are well established, and have been discussed in detail recently.^1,3^ Increasing evidence now indicates that cell–matrix adhesion complexes and their components are also essential regulators of endothelial barrier function. Landmark articles in the last decade have shown extensive involvement of integrin–matrix interactions, endosomal trafficking, and crosstalk between cell–matrix and cell–cell adhesions in endothelial barrier regulation. The signaling pathways that orchestrate cell–matrix adhesion may therefore provide novel and additional targets for therapies against vascular leak.

In this review, we discuss current insights into the role of cell–matrix complexes and their crosstalk with cell–cell junctions during blood vessel formation and remodeling, as well as in the resting endothelium and in (hemo)dynamic processes involving barrier alteration. In particular, we focus on mechanisms of adhesion turnover and reorganization in pathophysiological conditions with disturbed permeability like inflammation. Finally, we discuss potential future avenues for the development of therapeutic strategies to counteract endothelial dysfunction as a result of compromised cell adhesion.

## Diseases Associated with Endothelial Barrier Defects

Endothelial barrier dysfunction plays a central role in the pathophysiology of inflammatory conditions like sepsis and ARDS, as evidenced by preclinical and epidemiological studies. Interventions that attenuate endothelial barrier disruption strongly reduce mortality in animal models of sepsis or ARDS^4–6^; vice versa impairment of barrier-protective mechanisms has a deleterious effect on sepsis phenotype.^7^ Although direct measurement of vascular leak in patients remains challenging, clinical surrogate measures of vascular leak^8^ and circulating biomarkers of endothelial injury like Ang-2 (angiopoietin-2)^9^ directly relate to and predict mortality of sepsis and ARDS, independent of comorbidity.

Sepsis refers to a condition of infection accompanied by a systemic inflammatory response syndrome.^10^ Central in the development of sepsis is the damaging host response, characterized by systemic release of numerous cytokines. This cytokine storm invokes processes like endothelial activation, barrier disruption, and coagulation, which are responsible for the development of generalized vascular leak and systemic hypotension.^11^ Despite mortality rates ranging from 25% to 30% (severe sepsis) up to 40% to 70% (septic shock), the treatment is primarily supportive and lacks direct interventions.

ARDS is a syndrome characterized by severe and acute respiratory failure, resulting from injury to the alveolocapillary membrane with consequent pulmonary edema and impaired gas exchange.^12^ Etiological factors involve physical, chemical, or infectious hazards, such as high tidal volume ventilation, drowning, radiation, pancreatitis, acid aspiration, chemotherapy (eg, bleomycin), blood transfusion, and infection (sepsis or pneumonia). Within hours to days after exposure, alveolar edema and hypoxemic respiratory failure may develop, often requiring invasive mechanical ventilation. Increased endothelial permeability is a cardinal feature of ARDS, and many mediators and pathways contribute to disruption of endothelial barrier integrity.^12^ The central role of endothelial barrier disruption in ARDS is corroborated by studies showing that specific targeting of endothelial junctions can mimic alveolar edema in mice,^13^ whereas experimental manipulation of AJs^14^ or integrin-based adhesions^15^ in mice affects pulmonary vascular permeability. Much like sepsis, ARDS has high mortality rates.

The COVID-19 pandemic has resulted in a huge increase in the incidence of ARDS, as the majority of COVID-19 patients presents with acute hypoxemic respiratory failure as mono-organ failure. Autopsy studies revealed extensive injury to the capillary endothelium^16^ resulting from excessive cytokine production,^17^ giving rise to endothelial barrier disruption and in situ thrombosis.^17,18^ The role of the endothelium in COVID-19 has been reviewed elsewhere.^19^ In both sepsis and ARDS, intervention studies have primarily focused on restoring the dysregulated immune response, whereas studies testing intervention to improve endothelial integrity have been scarce.^20,21^

Viral hemorrhagic fever constitutes a group of viral infections in which the endothelial barrier is impaired, either due to direct damage to the endothelium or due to the host response leading to severe barrier disruption.^22^ In Ebola virus infection, EC toxicity and massive loss of ECs result in vascular permeability and hemorrhage.^23^ In contrast, infection with Hanta or Dengue virus leaves the endothelium intact, but invokes a strong pro-inflammatory response with release of inflammatory cytokines and Ang-2.^24^ Endothelial-specific mechanisms underlying subsequent barrier disruption involve among others neutrophil extracellular trap formation,^25^ sensitization to VEGF, and activation of the bradykinin–kallikrein system.^24^ Interestingly, different clinical syndromes cause vascular leak in different vascular beds, with predominant involvement of the visceral spaces in Dengue-virus–induced disease, and of the lungs in Hantavirus pulmonary syndrome.^22^ The central role of endothelial barrier dysfunction in these conditions with persistently high mortality rates illustrates the need for therapies that reverse endothelial barrier disruption.

Less-prevalent conditions include systemic capillary leak syndrome, anaphylactic shock, and Angiotensin-converting enzyme inhibitor-related and hereditary angioedema caused by reduced bradykinin degradation and C1-esterase inhibitor deficiency, respectively. Systemic capillary leak is a poorly understood condition characterized by episodes of generalized loss of endothelial barrier function without known pathogenesis, leading to systemic hypotension (shock) and generalized edema.^26^ Although the pathophysiology of this disease has not been clarified, plasma studies have identified surges of interleukin 6, VEGF, and Ang-2 during episodes of vascular leak.^26,27^ Endothelial barrier disruption takes place at a large scale in conditions like anaphylactic shock and C1-esterase inhibitor deficiency, in which bradykinin/kallikrein-mediated hyperpermeability plays a central role.^28^

## Endothelial Cell–Matrix Interactions in the Developing and Adult Vascular Bed

### The Adult Vascular Bed

**Figure 3.**
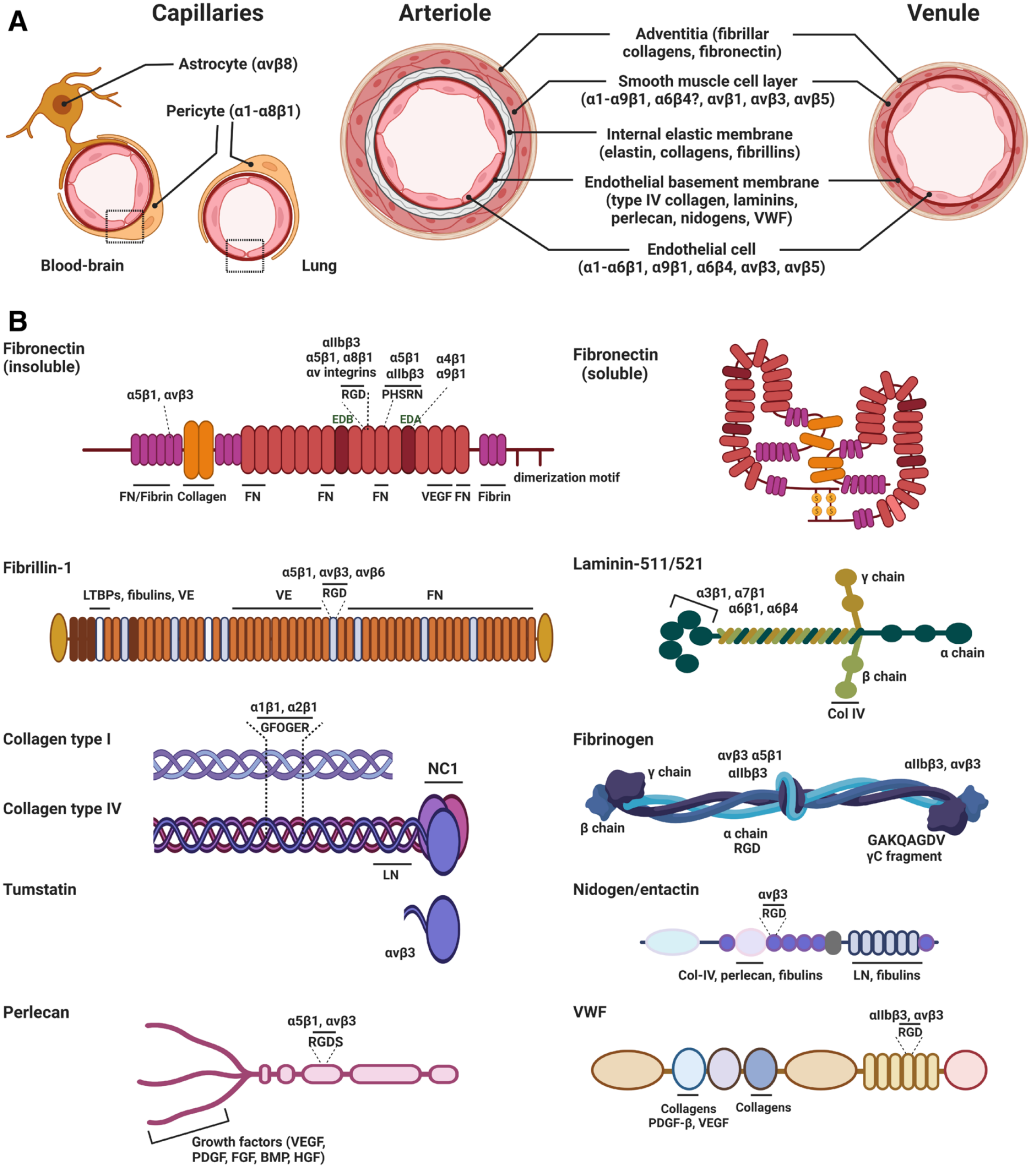
**Integrin–extracellular matrix (ECM) interactions in the blood vessel wall. A**, Main integrins and their ligands in the layers of blood vessels (see also Table 1). **B**, Schematic representation of some of the most common vascular ECM proteins and the main binding sites for integrins, growth factors, and other ECM molecules. Several more vascular ECM proteins are recognized by integrins, including decorin and versican. Integrins organize many matrix proteins into fibrils, such as collagen I (mainly integrins α11/α2β1) and fibronectin (FN; mainly integrin α5β1), and can also bind soluble forms of FN, vWF, and the γC fragment of fibrinogen. Many integrins recognize the RGD (Arginine-Glycine-Aspartate) sequence, for instance in fibrinogen, perlecan, vWF, fibrillins, and FN. In the endothelial basement membrane, laminins and collagen are abundant in quiescent endothelium. Upon injury, inflammation, or remodeling, the deposition of matrix and plasma proteins such as FN, vWF and fibrinogen is increased, as well as in provisional matrices associated with wound healing and angiogenesis. FN contains an RGD and PHSRN (also called the synergy site) motif. FN exists in several splice variants, and increased expression of the extra domain (ED)A and EDB isoforms of FN is associated with tissue remodeling. The variable region in FN, containing a binding site for α4 integrins, is also subject to alternative splicing and not depicted here. Note that the interaction between fibrillin and LTBP, which is responsible for regulated transforming growth factor (TGF)-β activation, is probably indirect. BMP indicates bone morphogenetic protein; Col, collagen; FGF, fibroblast growth factor; GFOGER, Glycine-Phenylanaline-Hydroxyproline-Glycine-Glutamic acid-Arginine; HGF, hepatocyte growth factor; LN, laminin; LTBP, latent transforming growth factor (TGF)-β binding protein; PDGF, platelet-derived growth factor; VE, versican; VEGF, vascular endothelial growth factor; and vWF, von Willebrand Factor. Created with Biorender.com.

The endothelial BM of fully mature, quiescent endothelium contains perlecan, laminins (mostly laminin-411 and -511), nidogens/entactins, and collagen type IV and XVIII, as well as von Willebrand Factor, which are synthesized and deposited by ECs and pericytes^29^ (Figure 3A and 3B). The presence and composition of the ECM is critical for vascular stability and function, as revealed by collagen IV- or perlecan knockout mice, which are characterized by vascular instability and hemorrhage.^30,31^ This may be due to both defective vessel formation and aberrant endothelial barrier regulation. Deletion of both nidogens in mice impaired BM formation and induced local microvascular leakage (mainly in the heart),^32^ while endothelial knockout of laminin-511 resulted in impaired response of arterial endothelium to shear stress.^33^ Damage to the endothelium results in the deposition of plasma proteins, including fibronectin, fibrin, fibrinogen, vitronectin, and von Willebrand Factor. Fibronectin is not abundantly present in the BM of quiescent endothelium, but the expression and synthesis of cellular fibronectin is markedly increased in angiogenic vessels during vascular remodeling, wound healing, or in pathological conditions, including atherosclerosis, tumors, fibrosis, and after myocardial infarction.^29^ In addition, BM proteins bind a variety of growth factors, including VEGF, angiopoietins, and proteins involved in the sequestration and activation of transforming growth factor β (Figure 3B), many of which can interact with integrins, in addition to their cognate receptors.

The expression of integrins in ECs generally follows the abundance of their cognate ligands in the BM; quiescent endothelium typically expresses the collagen-binding integrins α1β1 and α2β1, the laminin-binding integrins α3β1, α6β1, and α6β4, the fibronectin-recognizing integrins α4β1 and α5β1, the fibronectin/thrombospondin/tenascin receptor α9β1, and αvβ3 and αvβ5 (which together bind several RGD (Arginine-Glycine-Aspartate)-containing ligands, including vitronectin, fibronectin, nidogens, von Willebrand Factor, and fibrinogen; Table 1; Figure 3A and 3B).

**Table 1.**
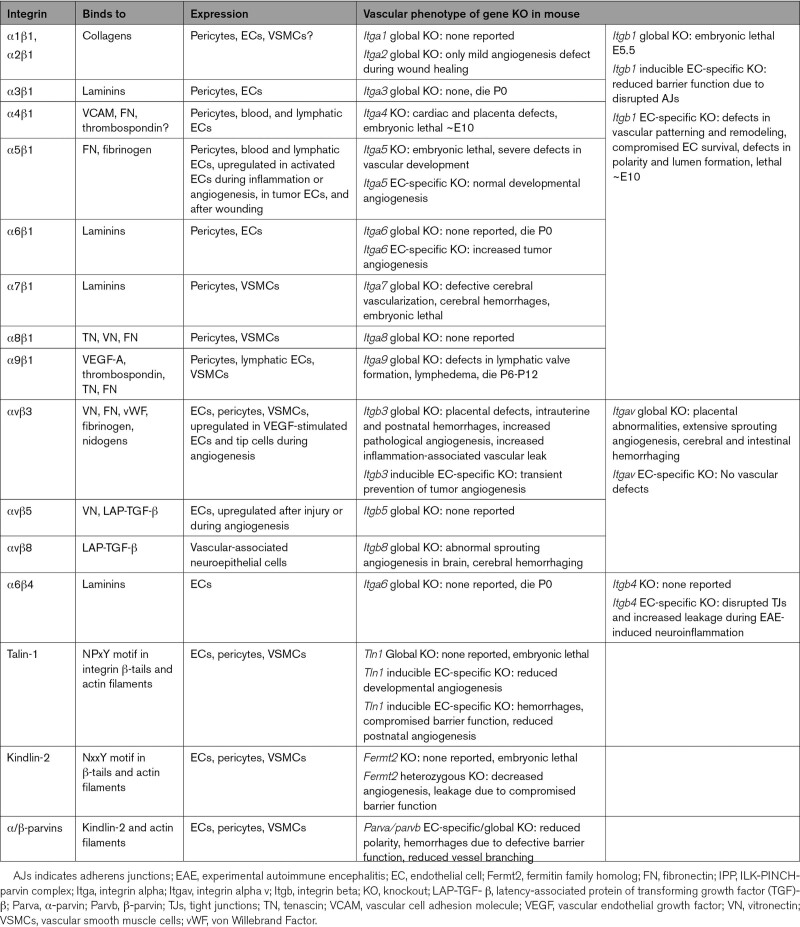
Integrins and Associated Proteins in the Vasculature and the Phenotypes of KO Mice

**Figure 4.**
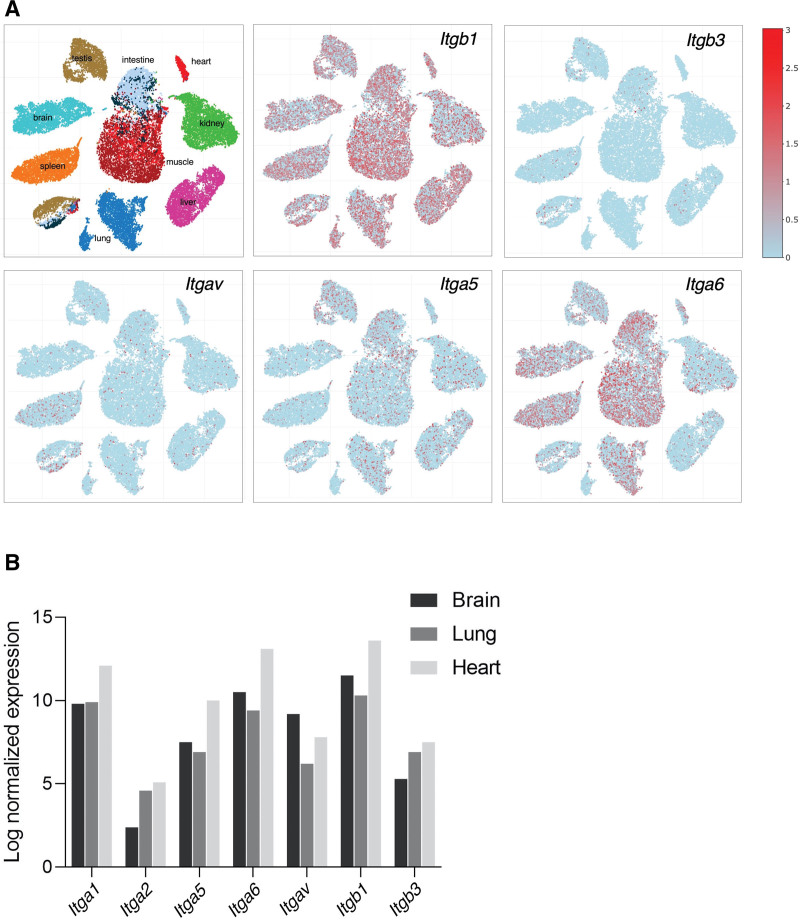
**Expression profiles of integrin subunits in various endothelia.** Data were obtained by in silico analysis of single-cell sequencing databases. Integrin subunit expression in various organs of adult mice, obtained from EC Atlas,; Carmeliet laboratory (https://endotheliomics.shinyapps.io/ec_atlas; **A**)^36^ and Rehman laboratory (http://rehmanlab.org/ribo; **B**).^35^

In addition, the expression of αvβ3 and αvβ5, as well as that of α5β1, is strongly upregulated in actively remodeling vessels during development, inflammation, and wound healing, in tumors, or in atherosclerotic plaques (Table 1; Figure 3A).^34^ Interestingly, expression of integrin α9β1 is mostly restricted to lymphatic ECs (Table 1). Although endothelial heterogeneity was conceptualized more than a decade ago, recent single-cell sequencing studies have fully elucidated the transcriptome of the different vascular beds in adult mice.^35,36^ Mining of these data indicates that the expression of most integrins is probably not very different across vascular beds (Figure 4). While little is known about vascular bed-specific roles of integrins in endothelial barrier integrity in the kidney, liver, and intestines, the role of integrins in blood-brain barrier integrity has gained significant attention. In cerebral ECs, β1 integrins contribute to blood-brain barrier integrity, as they are required for the localization of ZO-1 (zonula occludens-1) and claudin-5 to tight junctions.^37,38^ Moreover, the interaction of α5β1 with vitronectin is key in maturation of the blood-brain barrier, and EC-specific α5β1 deletion or mutation of the α5-binding site in vitronectin results in cerebral barrier disruption in mice.^39^ This is in contrast to the rest of the circulation, where α5β1 binds fibronectin and the presence of α5β1 or vitronectin signify immature BM or activated endothelium. Thus, with the abundant expression of common endothelial integrins in different vascular beds and sparce data on differential integrin function between different endothelia, there is currently little evidence for endothelial heterogeneity with regard to integrins in the adult endothelium under resting conditions. The brain may form an exception, as integrin regulation and function there may be different from that in other endothelia.

### Development and Angiogenesis

During development, several integrins cooperate to generate a functional vascular network (Table 1). Consistent with the expression of several β1 integrins in the vasculature, the endothelial-specific deletion of the β1-subunit in mice causes embryonic lethality due to severe defects in vascular development.^40,41^ This is not due to loss of collagen-binding integrins α1β1 and α2β1, as mice lacking either the α1 or the α2 subunit are viable and healthy and have no apparent vascular phenotype,^42^ although compensation between α subunits may occur here. Furthermore, whereas knockout of the gene encoding laminin α4 leads to excessive tip cell formation and sprouting due to dysregulated DLL4 (Delta Like Canonical Notch Ligand 4)/Notch signaling,^43^ the loss of individual laminin-binding integrins α3β1, α6β1, or α6β4 does not cause vascular defects, suggesting functional compensation.^42^ Deletion of the α4 subunit in mice results in embryonic lethality due to placental and cardiac defects.^42^ Interestingly, α4β1 promotes adhesion of ECs to vascular cell adhesion molecule-1 expressed on pericytes, and loss of this interaction results in apoptosis in both cell types, impairing blood vessel maturation and neovascularization.^44^ In addition, α4β1 interaction with fibronectin is needed for the uniform distribution of pericytes and vascular smooth muscle cells along cranial vessels.^45^ Integrin α9β1 binds several angiogenic regulators, including VEGF-A and thrombospondin, and is required for fibronectin fibril assembly by lymphatic ECs and for lymphatic valve morphogenesis. As a consequence, deletion of the α9 subunit in mice causes early postnatal lethality due to lymphedema in the pleural cavity.^46,47^

Consistent with the strong expression of fibronectin in developing vessels, fibronectin knockout in mice causes early embryonic lethality due to defects in cardiovascular development.^29^ Integrin α5β1 is the major receptor for fibronectin and is important for the reorganization of secreted fibronectin dimers into a fibrillar network, a process known as fibronectin fibrillogenesis. Consequently, defects in α5 integrin-null embryos are very similar to those in fibronectin-null animals, although somewhat less severe, and are aggravated by knockout of αv but not α4.^48^ The 5 αv integrins (αvβ1, αvβ3, αvβ5, αvβ6, and αvβ8) bind many ligands (Table 1) and therefore have complicated functions in the vasculature.^42^ Genetic ablation of the gene encoding αv in mice leads to embryonic death around mid-gestation likely due to placental abnormalities in 80% of αv-null embryos, while the remainder display dysregulated angiogenesis and die postnatally due to cerebral and intestinal hemorrhages.^49^ The defects in αv-null mice are largely attributed to the loss of αvβ8 because β8-deficient mice develop a similar phenotype, while deletion of either the β5 or the β6 subunit does not result in vascular defects.^50^ Furthermore, common genetic variants in the gene encoding β8 are associated with the occurrence of brain arteriovenous malformations in humans.^51^ Interestingly, these defects are due to the requirement of αvβ8 on parenchymal cells, including neuroepithelial cells, to activate transforming growth factor-β, which suppresses angiogenic sprouting and promotes vascular stability in the central nervous system.^52^ The β6 and β8 subunits, however, seem to be predominantly expressed on epithelial cells and immune cells, respectively, and are expressed at very low levels in the endothelium. The integrin αvβ3 is strongly expressed on angiogenic vessels, associates directly with VEGF receptor-2 (VEGFR2), and potentiates its activation. Numerous studies have shown that αvβ3 function-blocking peptides, antibodies or small molecule antagonists block VEGF-induced angiogenesis in several mammalian and avian model systems.^42,53^ However, while ablation of the β3-subunit in mice leads to embryonic death in about 50% of the animals due to fetal hemorrhage, the rest are born with postnatal hemorrhages and thrombocytopenia but no apparent vessel abnormalities, which is not due to compensation by αvβ5.^54^ Rather, β3 deletion leads to strong upregulation of VEGFR2 expression, which may be a feedback mechanism to rescue endothelial sensitivity to VEGF and results in enhanced VEGF-induced leakage and even increased pathological angiogenesis.^55^ Integrin αvβ3 can also bind anti-angiogenic ligands such as tumstatin, a fragment of the noncollageneous domain of the Collagen-IV α3 chain found in the BM and provisional matrices (Figure 3B), which is known to block angiogenesis by inducing EC apoptosis.^56^ Therefore, the role of αvβ3 is complicated and seems to be dependent on VEGFR2 expression and available ligands, as well as crosstalk with other endothelial integrins, as will be detailed below. Importantly, pathways regulating angiogenesis may vary between tissues, and pathological angiogenesis in the adult may depend on factors other than those regulating developmental angiogenesis. During hypoxia-induced blood vessel remodeling in the spinal cord and the brain, as well as in tumor angiogenesis, several integrins and integrin ligands are upregulated, which also play a role in physiological angiogenesis, in particular integrins αvβ3, α5β1, and fibronectin.^42,57,58^ Hypoxia-induced expression of these integrins, as well as VEGF, is regulated by transcription factors of the HIF (hypoxia-inducible factor) family, including HIF-2α, and ECs derived from HIF-2α-null mice display low expression of αvβ3, α5β1, and fibronectin, which is associated with reduced cell adhesion and impaired tumor angiogenesis in vivo.^59^ While HIFs also induce VEGF expression during hypoxia, integrin αvβ3 can regulate VEGF expression at the posttranscriptional level through eukaryotic initiation factor 4E protein under normoxic conditions, which is inhibited by αvβ3 binding to tumstatin.^56,60^ There are also reports that show a role for collagen-binding (α1β1, α2β1) and laminin-binding (α3β1, α6β1) integrins in postischemic or tumor angiogenesis.^61^ The role of these integrins may be highly context-specific and involve nonclassical functions; for instance, α3β1 was found to repress angiogenesis into subcutaneous tumors and hypoxia-induced retinal angiogenesis by regulating endothelial VEGF levels,^62^ whereas in another study α3β1 enhanced angiogenesis in glioblastoma by promoting macropinocytosis and lysosomal exocytosis, important for lumen formation in nascent vessels.^63^

Altogether, integrins are abundantly expressed in the endothelium of developing and mature, adult vasculature. While there is currently little knowledge of variation in expression of specific integrins between the vascular beds of various organs, integrin expression is strongly influenced by inflammation, hypoxia, angiogenesis, and wounding. Moreover, integrin function is strongly determined by context-specific clues, including the presence of available ligands and integrin-binding partners, the physical properties of the vascular bed, and signaling pathways determining spatiotemporal subcellular distribution, as will be detailed below.

## Dynamic Regulation of Endothelial Cell–Matrix Adhesion in (Patho)Physiological Conditions Like Angiogenesis, Inflammation, and Shear Stress

### The Composition of Cell–ECM Adhesions

FAs contain a wide variety of structural, signaling, and adapter proteins. Some FA components bind directly to integrin cytoplasmic tails, such as talins and kindlins, which recognize the conserved NPxY/NxxY motifs in integrin β-subunits through their FERM (protein 4.1, ezrin, radixin, moesin) domains^64^ (Figure 2C). Talin and kindlin binding increases the affinity of integrins for their ligands, a process called integrin activation (Figure 2C), and is effectuated by an inside-out mechanism involving the GTPase Rap1 (Ras-related protein 1), in response to signals emanating from G-protein–coupled receptors or growth factor receptors.^64^ Downstream of integrin-ligand binding, talins and kindlins also enhance integrin clustering and their linkage to the cytoskeleton, and are essential for cell spreading.^64^ Not surprisingly, ablation of the genes encoding these proteins produces similar defects as integrin knockouts. For instance, endothelial talin-1 ablation results in embryonic lethality in mice due to defects in formation of the vasculature, including dilation and poor branching.^65^ Similarly, compromised endothelial expression of kindlin-2 induces vascular leakage and angiogenic defects in mice, and is associated with reduced pericyte content and defective vessel maturation.^66^ Intriguingly, kindlin-2 resides not only in FAs but also in AJs,^67^ and may thus directly regulate barrier function by affecting the formation, maintenance or stability of AJs. In addition to kindlin-2, several other proteins have been identified in both integrin- and cadherin-based adhesion complexes, among which kinases such as focal adhesion kinase (FAK), c-Src (Proto-oncogene tyrosine-protein kinase Src)- and Abelson (Abl)-family kinases, adapter and cytoskeletal scaffold proteins such as vinculin, filamin, and migfilin, and actin-associated proteins, including α-actinins, VASP (vasodilator-stimulated phosphoprotein), and zyxin^67^ (Table 2).

**Table 2.**
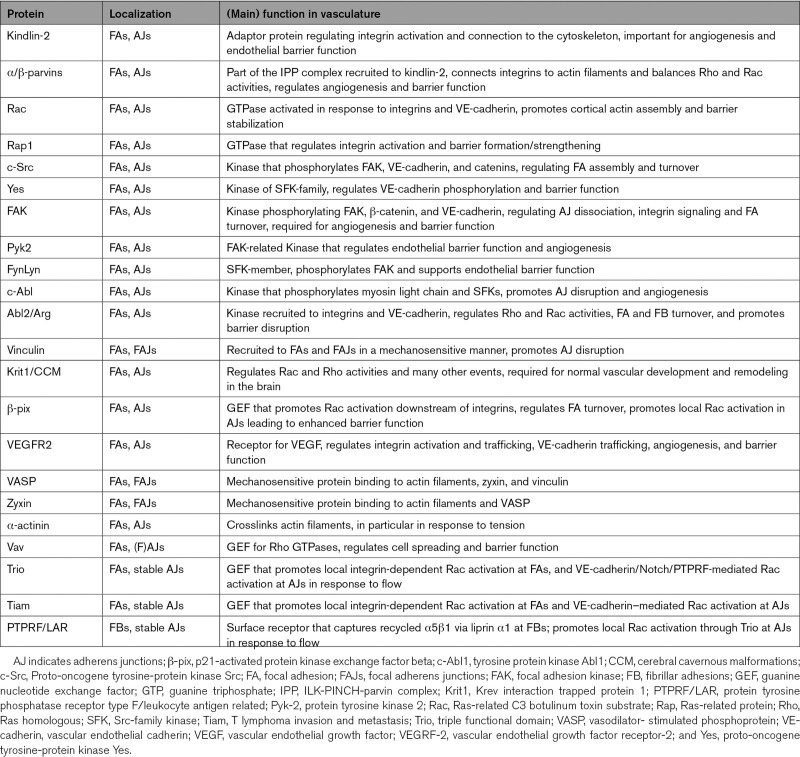
Main Proteins Found in both Cell–ECM and Cell–Cell Junctions in ECs and Their Function

Strikingly, many of these components impact on vascular permeability, either through direct effects on adhesion components (eg, phosphorylation), or through long-range biochemical signaling or mechanical signaling. Kindlin-2 recruits a tripartite complex to FAs consisting of ILK (integrin-linked kinase), PINCH (particularly interesting cys-his-rich protein), and parvins (Figure 2C). Endothelial deletion of α-parvin perturbs both cell–matrix and cell–cell adhesions, leading to angiogenesis and barrier defects in mice, which is at least partially due to differences in Rac and Rho activities.^68^ A similar phenotype is seen in endothelial ILK knockout mice, while in humans, mutations in ILK have been disclosed in familial exudative vitreoretinopathy.^69^

### Regulation of Cell–ECM Adhesion by Protein Kinases

Kinases play a central role in the dynamic regulation of cell adhesion. In the resting endothelium, many kinases reside in both cell–cell junctions and cell–matrix adhesions. While the role of endothelial kinases in AJ regulation has recently been reviewed,^3^ we will focus here on their role in cell–matrix adhesion.

FAK was identified early on as a member of FAs, and its involvement in endothelial barrier regulation under inflammatory conditions is complex and agonist-specific.^70^ FAK binds to paxillin and talin, and is activated by autophosphorylation upon ECM binding or by phosphorylation by SFKs (Src-family kinases) in response to barrier-disruptive stimuli. Endothelial FAK knockout in mice results in early embryonic mortality due to decreased EC survival, vascular instability, and impaired angiogenesis.^71,72^ While kinase-independent functions underlie FAK’s involvement in vascular stability during development, the kinase function of FAK is required for endothelial barrier maintenance in adult life in mice.^73^ Studies in human and bovine pulmonary arterial ECs, HUVECs, intact perfused coronary venules, and in mice have shown that the role of FAK activation in response to barrier-disruptive agents is highly dependent on phosphorylation of specific sites within FAK, its subcellular localization, and the temporal dynamics of the barrier-disruptive response. After exposure to thrombin or VEGF, FAK accumulates in elongated FAs,^74,75^ which involves RhoA activity and FAK phosphorylation on tyrosines 397, 576, and 925.^74^ The initial contractile phase is followed by a phase of relaxation, during which p190 RhoGAP (p190 Ras homology GTPase activating protein) is phosphorylated by FAK, resulting in RhoA inactivation.^76^ During barrier recovery, or after stimulation with barrier-enhancing agents like sphingosine-1-phosphate, FAK is found in punctate peripheral FAs.^77^ This phase is characterized by c-Src-dependent FAK phosphorylation at residue Y576,^74^ and parallels activation of Rac1, the formation of a cortical actin belt, and cell spreading. Together, these studies demonstrate a dual involvement of FAK in endothelial barrier regulation, with an initial contribution to the agonist-induced contractile response in parallel with increased RhoA activity, followed by RhoA inactivation and subsequent barrier recovery with peripheral relocation of FAs and cell spreading. Apart from its localization in FAs, FAK localizes to AJs in HUVECs and in mouse heart ECs, where it binds the cytoplasmic tail of VE-cadherin and contributes to AJ dissociation by phosphorylation of β-catenin (Y142).^78^

SFKs are activated downstream of a wide array of barrier-disruptive agents, in particular VEGF-A/VEGFR2 stimulation. Classically, the SFK pool residing in cell–cell junctions mediates barrier disruption via tyrosine phosphorylation of AJ proteins, including VE-cadherin in cultured ECs and in mice.^79,80^ Depending on the residue, VE-cadherin phosphorylation may lead to internalization, a process that is constitutively suppressed in a resting monolayer by tyrosine phosphatases.^81,82^ An exception within the SFK-family is Lyn, which rather supports the endothelial barrier through a mechanism involving the phosphorylation of FAK (at Y576, Y577, and Y925).^83^ A second pool of c-Src is found in FAs,^84^ where it regulates actin and adhesion dynamics. The exact function of c-Src in FAs is less well understood, among others due to complex and bidirectional interactions with FAK. In cultured ECs, c-Src phosphorylates FAK and paxillin, which drives FA assembly, and regulates angiogenic sprouting in mice.^85^ Loss of c-Src resulted in reduced matrix adhesion and sprout stability. As the effect of c-Src on angiogenic sprouts was observed without an effect on endothelial barrier integrity, this study implies that spatially separate pools of c-Src may have distinct effects on cell–cell junctions and cell–matrix adhesions. There is evidence that modulation of integrins, for example by varying matrix stiffness or integrin-modulating antibodies, can signal via FAK and c-Src to affect cell–cell junction integrity,^71,86^ indicating crosstalk between cell–matrix adhesions and cell–cell junctions in endothelial barrier regulation.

Arg (Abl-related gene)/Abl2 is a nonreceptor tyrosine kinase that associates with integrins and is a negative regulator of integrin function.^87^ Arg/Abl2 can be activated by inflammatory mediators and other barrier-disruptive agents, including thrombin, VEGF, histamine, TNF-α, and lipopolysaccharide.^88–91^ Arg/Abl2 promotes agonist-induced endothelial barrier disruption, as its depletion or pharmacological inhibition attenuated barrier disruption, in HUVECs and human pulmonary microvascular ECs in vitro, as well as in mice.^88,91^ Depletion of Arg/Abl2 reduced FA disassembly and increased the number of FAs, particularly in the cell periphery.^91^ This was paralleled by enhanced cell spreading and improved adhesion to the ECM, which may be explained by the observed increase in Rac1 or Rap1 activity.^88,89,91,92^ Arg depletion also resulted in elevated RhoA activity, which may be due to the fact that Arg phosphorylates and activates p190RhoGAP.^93^ Interestingly, the increase in actomyosin contraction upon Arg depletion does not result in barrier disruption, suggesting a parallel increase in tethering force withstanding cell contraction, probably mediated by the increased cell–matrix adhesion and spreading. While these effects were observed upon stimulation with thrombin and histamine, knockdown of Arg attenuated lipopolysaccharide-induced endothelial barrier disruption via reduced MLC (myosin light chain) phosphorylation,^94^ indicating that Arg-activated signaling pathways may differ depending on the vasoactive agent. Abl1/c-Abl also mediates endothelial barrier disruption, via phosphorylation of caveolin-1 upon stimulation by oxygen radicals,^95^ and via its action on the cortical actin belt, by direct phosphorylation of MLC (residues Y231, Y464, Y556, and Y846) and paxillin.^96,97^ After stimulation with VEGF, c-Abl is activated downstream the Neuropilin-1 cytoplasmic domain to activate SFKs, resulting in vascular permeability and angiogenesis.^98^ In contrast to Arg, no effects of c-Abl on cell–matrix adhesion have been reported.

The serine/threonine kinase Mitogen-activated Protein Kinase kinase kinase kinase (MAP4K4) is also involved in FA turnover. Depletion of MAP4K4 in HUVECs resulted in decreased FA disassembly and reduced cell motility during angiogenesis.^99^ MAP4K4 phosphorylates the FERM domain of moesin, which then competes with talin for binding to the β1 integrin tail, thereby inactivating β1 integrins. Knockdown of either MAP4K4 or moesin reduced FA disassembly, and impaired HUVEC sprouting and retinal angiogenesis in mice.^99^ Under inflammatory conditions, pharmacological inhibition or endothelial deletion of MAP4K4 in mice attenuated endothelial barrier disruption by TNF-α or thrombin.^100,101^ Also during endothelial barrier disruption, MAP4K4 signaled via ezrin/radixin/moesin to increase the turnover of FAs, leading to enhanced inter-endothelial gap formation.^101^

### Regulation of Cell–ECM Adhesion by Endosomal Trafficking

Accumulating evidence indicates a major role for the endolysosomal system in the formation and dissolution of both integrin- and cadherin-based cell adhesions. Intracellular transport along endolysosomal routes requires specialized protein complexes residing in distinct compartments that mediate the fusion of membranes, cargo sorting, and transport along the cytoskeleton.^102^ The involved cellular machinery typically consists of GTPases of the Rab/Arf families, their GAPs (GTPase-activating proteins), GEFs (guanine nucleotide exchange factors) and effectors, and motor proteins. In addition, tethering complexes bring vesicles into close proximity with target membranes, while subsequent membrane fusion is mediated by SNARE (Soluble NSF Attachment REceptor) proteins. There is great diversity in the endolysosomal system; over 60 different SNAREs and over 70 different Rab GTPases have been identified. While a detailed description of the endosomal trafficking machinery is beyond the scope of this review, we will here discuss recent insights that are relevant for integrin function in ECs.

**Figure 5.**
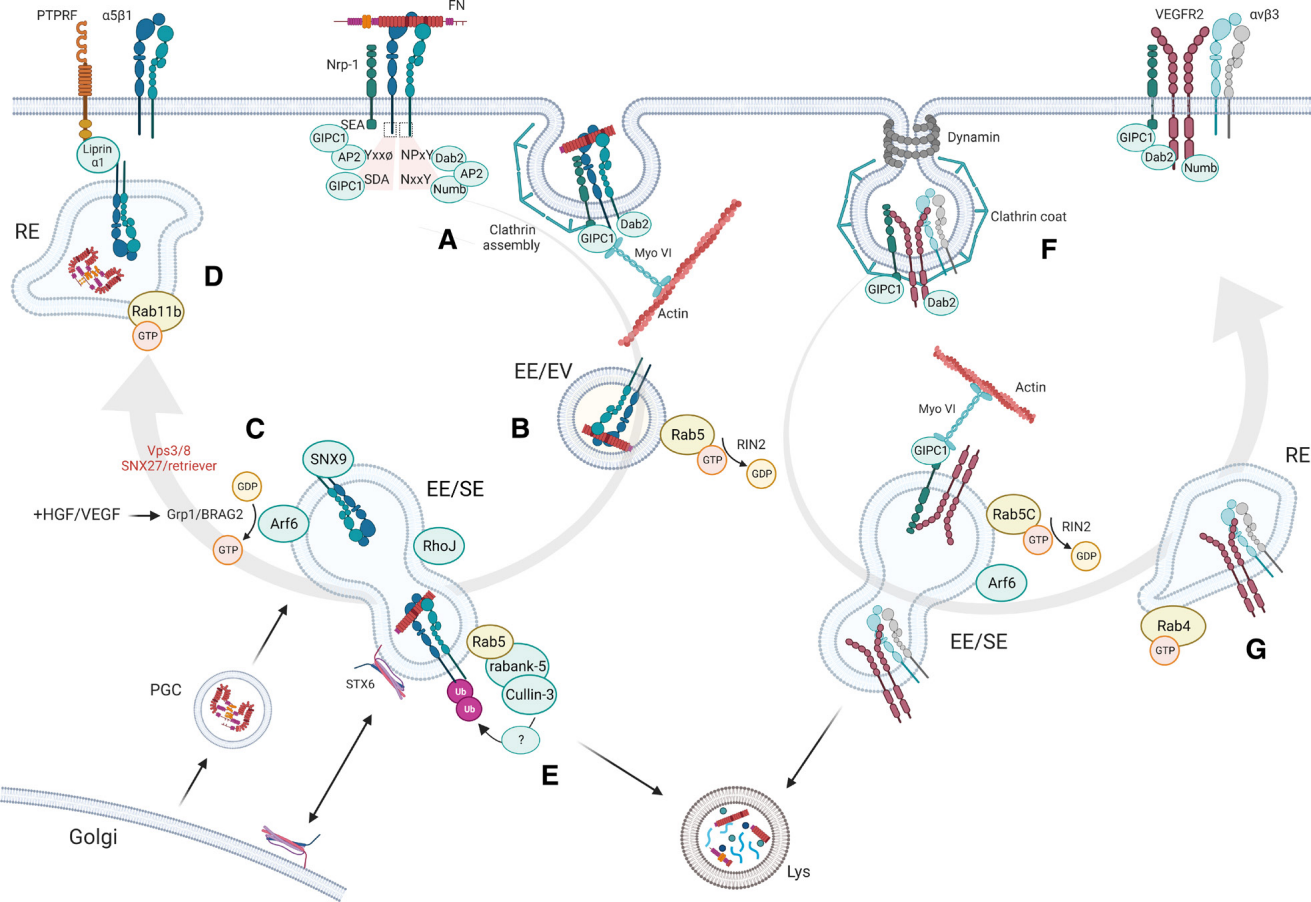
**Endosomal traffic of integrins and associated proteins in endothelial cells (ECs). A**, Integrins can be internalized by caveolin- or clathrin-dependent endocytosis, mediated by several adaptor proteins (Dab2, Numb, AP2, GIPC1) that bind to motifs in integrin cytoplasmic tails (NPxY/NxxY in the β-tail, YxxΦ and SDA in some α-tails such as α5). Integrins can also be endocytosed in combination with other receptors; for example α5β1 co-internalizes with neuropilin-1 (Nrp-1), mediated by GIPC1 and myosin-VI (Myo VI). **B**, Internalized integrins and associated proteins are delivered to early endosomes (EEs) in a RIN2/Rab5 dependent manner. **C**, In EEs, sorting toward the recycling route takes place under control of several factors, including RhoJ, syntaxin-6 (STX6), sorting nexin-9 (SNX9), and Arf6, whose activation is stimulated by HGF and VEGF. Factors indicated in red have been identified as regulators of integrin trafficking in other cell types than ECs. **D**, Some recycling pathways combine with the biosynthetic route to deliver de novo synthesized FN from the Golgi together with recycled α5β1 to the cell-surface, where it is captured by liprin-α1. **E**, Internalized integrins can also be routed to lysosomes for degradation. **F**, VEGFR2 can exist on the cell-surface in complex with several other receptors, including integrins and Nrp-1, and follows similar routes as integrins. VEGFR2 internalization is promoted by Dab2 and Numb, as well as GIPC1 binding to Nrp-1, and myosin-VI mediates transport along actin filaments to EEs. Protection from degradation depends on RIN2/Rab5C, and recycling is promoted by Nrp-1 via Rab11+ vesicles, or via Rab4+ vesicles, where VEGFR2 co-traffics with integrin αvβ3 (**G**). Despite the identification of many individual components, a comprehensive view of how and where all of these components interact is largely lacking. In addition, while for many of these factors a role in protein trafficking during angiogenesis has been established, their role in barrier function remains less clear. AP2 indicates adaptor protein 2; Arf, ADP ribosylation factor; BRAG2, brefeldin A-resistant ArfGEF 2; Dab2, disabled-2; EE, early endosome; EV, endocytic vesicle; FN, fibronectin; GDP, guanine diphosphate; GIPC, GAIP interacting protein, C terminus; Grp1, general receptor for 3-phosphoinositides 1; GTP, guanine triphosphate; HGF, hepatocyte growth factor; Lys, lysosome; Myo VI, Myosin-VI; NPxY, Asparagine-Proline-x-Tyrosine; NxxY, Asparagine-x-x-Tyrosine; PGC, post-Golgi carrier; PTPRF, protein tyrosine phosphatase receptor type F; Rab4, Ras-like protein in brain 4; Rab5, Ras-like protein in brain 5; Rabank-5, rabankyrin-5; RE, recycling endosome; RhoJ, Ras homologous J; RIN2, Ras And Rab Interactor 2; SDA, Serine-Aspartic acid-Alanine; SE, sorting endosome; SNX27, Sorting Nexin 27; Ub, ubiquitin; VEGF, vascular endothelial growth factor; VEGFR, vascular endothelial growth factor receptor; and vps3, vacuolar protein sorting 3. Created with Biorender.com.

Integrin internalization can occur via caveolae, macropinocytosis, or clathrin-coated pits. Clathrin-dependent integrin endocytosis is regulated by sorting signals in integrin cytoplasmic tails, including an YxxΦ motif found in a number of α-subunits, and the NPxY/NxxY motifs in β-subunits, which serve as canonical signals for adaptor proteins that recruit cargo into clathrin-coated pits.^103,104^ A number of well-known clathrin adaptors that bind NPxY/NxxY motifs, including Dab-2 (Disabled-2), Numb, and AP-2 (adaptor protein-2), promote FA disassembly^104^ (Figure 5A). Consistent with the importance of endocytosis for cell migration, the endothelial deletion of Dab-2 or Numb impairs or delays angiogenesis in mice.^105,106^ However, Numb and Dab-2 interact with a range of other cell-surface proteins (including VEGF receptors) and regulate VEGF signaling. It is therefore likely that the inhibition of angiogenesis observed in the absence of Numb or Dab-2 reflects their combined effects on integrins, VEGF receptors, and other pro-angiogenic molecules. Knockdown in HUVECs of dynamin-2, a GTPase required for the fission of endocytic vesicles, also impairs integrin endocytosis and FA disassembly, and endothelial deletion of dynamin-2 disturbs developmental angiogenesis in mice. Although dynamin-2 mediates endothelial barrier disruption, these effects were attributed to reactive oxygen generation.^107^ VEGF signaling is augmented in the absence of dynamin-2, probably because of VEGFR2 accumulation at the cell-surface due to impaired endocytosis. Thus, dynamin-2 disconnects VEGF signaling from VEGF-induced migration through integrins during angiogenesis.^108^

Internalized integrins are delivered to early endosomes (EEs; Figure 5B). Here, cargo is collected and sorted under the control of Rab5 GTPases (Rab5A,B,C and related Rabs such as Rab21), some of which can bind directly to integrin cytoplasmic tails and are required for integrin-dependent cell adhesion and migration.^104^ In HUVECs and in zebrafish, Rab5C (Ras-like protein in brain 5C) is the main Rab5 family member driving vascular development, in part because it maintains VEGF signaling by blocking VEGFR2 degradation.^109^ Additional studies in HUVECs have shown that the Rab5-GEF RIN2 (Ras and Rab Interactor 2) is required for Rab5C-dependent VEGFR2 maintenance and sprouting angiogenesis,^109^ and moreover promotes the selective endocytosis of active, ligand-bound α5β1 from fibrillar adhesions.^110^ This process is in human umbilical artery ECs also regulated by a complex of Neuropilin-1 and GIPC1 (GAIP-interacting protein, C-terminus)/synectin, a protein that interacts with the integrin α5-tail through its PDZ (post synaptic density protein, Drosophila large disk tumor suppressor, and zonula occludens 1) domain^111^ (Figure 5B). In pulmonary artery endothelial cells, the pro-inflammatory cytokine TNF-α regulates internalization of α5β1, leading to reduced presence of α5β1 integrins in FAs, reduced ECM adhesion, and enhanced endothelial permeability.^112^ This was partially reversed by fibronectin or an α5β1-activating antibody.^113^

In addition to Rab5, Arf6 (ADP ribosylation factor 6) is emerging as an important EE-resident GTPase that regulates integrin traffic in ECs. Arf6 drives endocytic recycling of β1 integrins in response to hepatocyte growth factor, and promoted adhesion and migration of immortalized murine ECs in vitro. In mice, Arf6 knockout in vascular ECs did not affect developmental angiogenesis, but reduced neo-angiogenesis in grafted tumors.^114^ Activation of Arf6 downstream of MAP4K4 promotes integrin internalization, and its activation by VEGF-mediated endothelial hyperpermeability and vascular instability.^115,116^ However, deletion of Arf6 in lymphatic endothelial cells caused severe edema and disruption of lymphatic vasculature in mice, underscoring the importance of Arf6 in lymphatic barrier function and lymphangiogenesis.^117^ Upstream of Arf6, 2 GEFs have been identified (Grp1 [General receptor for 3-phosphoinositides 1] and Brag2 [Brefeldin A-resistant ArfGEF 2]) that are required for Arf6-mediated β1 integrin endocytosis and sprouting angiogenesis under the control of hepatocyte growth factor and VEGF^117,118^ (Figure 5C).

Most internalized integrins are recycled back to the plasma membrane, which is dependent on integrin cytoplasmic tail sequences. Several factors involved in β1 integrin recycling have been identified in cell types other than ECs. Rab5 recruits the class C CORe Vacuole/Endosome Tethering (CORVET) complex, which mediates the homotypic fusion of EEs and cargo sorting. CORVET components can also stimulate the transit of β1 integrins from EEs to recycling endosomes, and thereby influence their return to the cell surface^119^ (Figure 5C). Recycling of β1 integrins requires an intact NxxY motif,^120,121^ recognized by the FERM domain of sorting nexin-17, which promotes sorting of β1 integrins toward recycling endosomes that are marked by Rab11 GTPases (Figure 5C). While the role of sorting nexin-17 in ECs remains to be affirmed, knockdown of sorting nexin-9 inhibited β1 integrin recycling in HUVECs, thus compromising β1 surface expression and disrupting cell spreading and tube formation.^122^ Strikingly, knockdown of the majority of FERM domain-containing sorting nexins did not disrupt tube formation. It remains to be determined which sorting nexins are necessary for β1 integrin recycling in ECs, and whether direct interaction with the β1 tail is required. The endosomal GTPase RhoJ and the PDZ-domain-containing polarity protein Scribble also regulate α5β1 recycling (Figure 5C).^123,124^ Scribble interacts directly with the α5 tail and blocks α5β1 translocation to lysosomes and its subsequent degradation. Silencing of Scribble reduced both cell-surface and total protein levels of α5, impaired HUVEC migration and spheroid outgrowth in vitro, and delayed the formation of intersegmental vessels in transgenic zebrafish embryos.^123^

Finally, Rab11b promotes the recycling of active, fibronectin-bound α5β1 in umbilical arterial ECs.^125^ The latter pathway also requires the ubiquitous adaptor liprin α1, which associates with the integrin β1 tail, and combines recycling of active α5β1 with transport of newly synthesized fibronectin dimers in post-Golgi carriers, leading to basolateral fibronectin secretion and fibrillogenesis (Figure 5D). Defects in integrin recycling, induced either by the absence of recycling proteins or by integrin cytoplasmic tail mutations that prevent their recruitment, cause mis-sorting of integrins to lysosomes and their subsequent degradation.^120,121^ Nevertheless, a fraction of internalized and fibronectin-bound α5β1 is actively directed to the lysosome, which is regulated by ubiquitination of α5β1 cytoplasmic tails.^126^ While the involved ubiquitin ligases and associated machinery are only beginning to be explored, the early endosomal factors cullin-3 and rabankyrin-5 are crucial to maintain integrin β1 surface levels in HUVECs, which is important for angiogenesis^127^ (Figure 5E). The SNARE protein syntaxin-6, which is localized in the Golgi network and EEs, is also required for integrin recycling in HUVECs, as the loss of syntaxin-6 leads to increased integrin ubiquitination and misrouting to lysosomes.^128^

It is noteworthy that integrin traffic is strongly influenced by their associations with other receptors including neuropilins as described above, and VEGFR2 (Figure 5A and 5F), which can exist in complex with β1 integrins and αvβ3.^129,130^ VEGFR2 association with β1 integrins promotes signaling induced by matrix-bound VEGF,^129^ while VEGFR2/αvβ3 interactions enhance reciprocal activation of both receptors and downstream signaling, as well as joint traffic through EEs and the Rab4-dependent short-loop recycling pathway (Figure 5G).^130^ Many other factors regulate traffic of both VEGFR2 and certain integrins in ECs, including Dab-2, Numb, GIPC1, MyoVI (Myosin VI), Rab5C, RIN2, and Arf6, but it remains to be seen whether these factors specifically act on VEGFR2/integrin complexes or rather are generic regulators of endosomal traffic.

These observations highlight the importance of integrin trafficking machinery for endothelial migration and survival, and show its dynamic regulation during endothelial exposure to inflammatory cytokines. Yet, it remains largely unknown how integrin trafficking is involved in endothelial barrier regulation mainly because a comprehensive and integrated view of the composition and interactions of all protein complexes involved is still lacking.

### Crosstalk Between Cell–ECM and Cell–Cell Adhesions

The targeted deletion of a number of integrin subunits in mice induces vascular leakage and hemorrhage as a result of blood vessel destabilization. Also during inflammation, integrins play a central role in the dynamic regulation of barrier function, in a complex and reciprocal interplay with several GTPases. In a number of in vitro model systems (including human pulmonary ECs, HUVECs, and intact rat kidney arterioles), barrier-disruptive agents (including thrombin, histamine, and VEGF) induce a rapid and transient increase in permeability by increasing RhoA activity, leading to a Rho-associated kinase (ROCK)-dependent increase in MLC phosphorylation and resulting actin cytoskeletal contractility.^131–133^ Pulling forces by actin filaments stimulate the formation and growth of FAs and fibrillar adhesions^134^ and transform stable AJs into remodeling junctions with a zipper-like appearance designated focal AJs (Figure 2B), which contain the mechanotransducer vinculin and are associated with a transient increase in permeability.^135^ RhoA/ROCK-induced contractility promotes histamine-stimulated focal AJ formation and anaphylactic shock in mice, and inhibition of this pathway protects against vascular leakage and anaphylaxis.^136^ The thrombin-induced increase in RhoA activity is paralleled by inactivation of Rac1, as well as inhibition of cAMP (cyclic adenosine monophosphate) signaling to Epac (exchange protein activated by cAMP) and Rap1 thus counteracting barrier-protective mechanisms.^137^ During the subsequent recovery phase, Rac-mediated cell spreading allows the re-establishment of nascent adhesions and AJs, whereas RhoA activity is suppressed, which can occur through p190RhoGAP or Arhgap29 (Rho GTPase Activating Protein 29). Interestingly, the latter interacts with the Rap1 effector Rasip1, and depletion of Arhgap29 leads to increased RhoA activity and decreased Cdc42 and Rac1 activities, strongly compromising the formation of AJs.^138^ Thus, the timed activation and inactivation of several different GTPases regulates both integrin-dependent and VE-cadherin–dependent adhesion complexes. In turn, the GTPases are regulated by these complexes. VE-cadherin can support activation of both Rac and RhoA, and this can feedback onto cell–matrix adhesions. For instance, upon contact inhibition in pulmonary artery EC monolayers, VE-cadherin inhibits cell spreading by activating RhoA.^139^ Since VE-cadherin antagonizes VEGFR2 signaling, inhibition of VE-cadherin/RhoA/ROCK signaling stimulates VEGF-driven sprouting through Rac1, both in HUVECs and in zebrafish.^140^

Recent studies show that integrins are profoundly involved in the response to barrier disruption. Endothelial deletion of β1 integrins or talin in mice leads to disruption of AJs and increased VE-cadherin internalization,^141,142^ and β1 integrins are required for the localization of ZO-1 and claudin-5 in the blood-brain barrier.^37,38^ Although this indicates a general role for β1 integrins in stabilizing the cell–cell junctions, it is also becoming clear that some β1 integrins, most notably α5β1, have antagonizing effects on endothelial barrier function during inflammation, which seems partially dependent on differential activation of Rho GTPases. In HUVECs and blood microvascular ECs, integrin α5β1 strongly promotes or sustains RhoA activation and cytoskeletal contractility, leading to disruption of AJs and intercellular gap formation.^143–145^ Moreover, blocking antibodies against β1 or endothelial haploinsufficiency of β1 protects against lipopolysaccharide-induced inflammation and vascular leakage in mice.^145^

**Figure 6.**
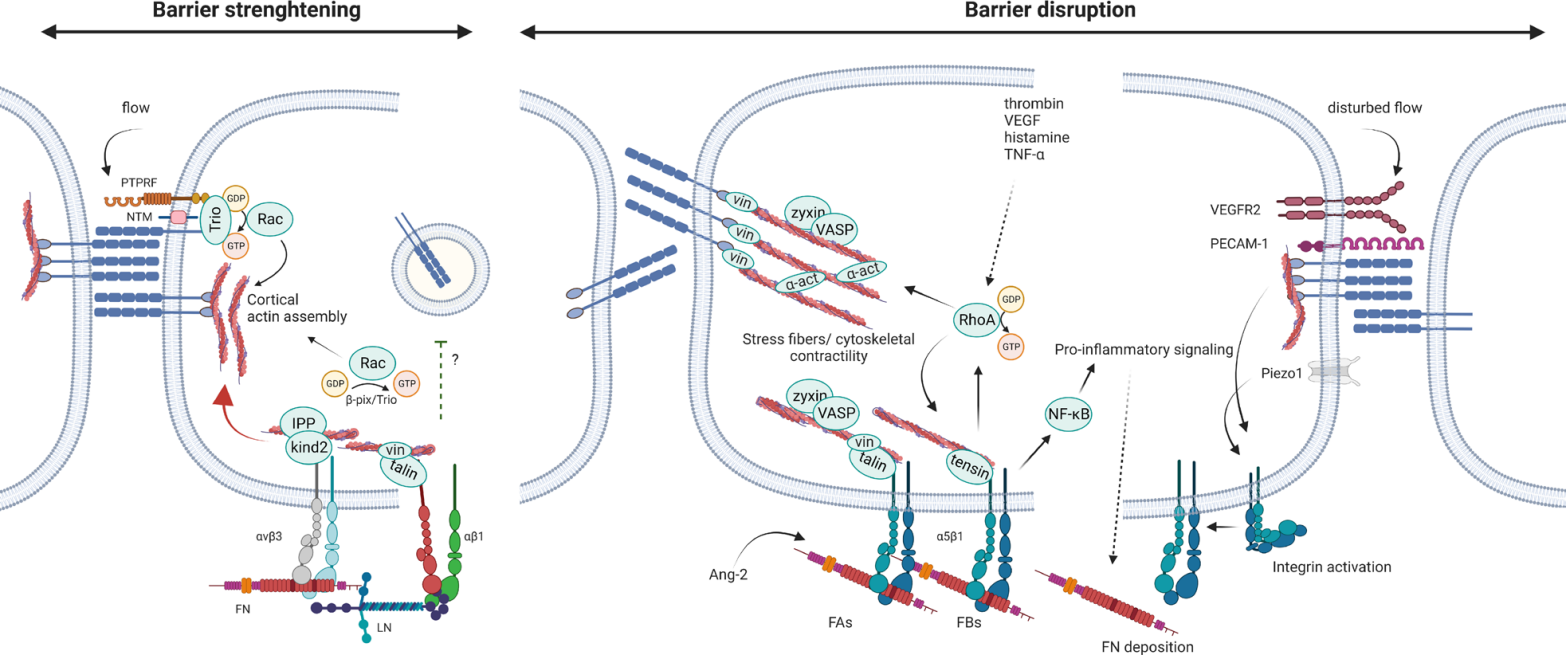
**Crosstalk between cell–extracellular matrix (ECM) and cell–cell complexes in endothelial barrier function.** FAs and AJs share many common components, including GTPases and their GEFs (guanine nucleotide exchange factors), adaptor proteins, actin-associated proteins, and several kinases (see also Table 2). Integrin αvβ3 and several β1 integrins can strengthen cell–cell junctions such as AJs (red arrow), although the mechanisms remain poorly understood. Integrins maintain overall cellular architecture and increase global Rac activation, leading to cortical actin assembly and strengthening of AJs. Rac activation is also locally increased at AJs in response to flow, by a complex including vascular endothelial cadherin (VE-cadherin), PTPRF, Notch, and the Rac GEF Trio, which also promotes Rac activation from FAs. In the absence of β1 integrins there is increased presence of VE-cadherin in vesicles but it is unclear if cell–ECM complexes actively repress VE-cadherin internalization or whether this is merely an effect of altered barrier properties. During barrier-disruptive conditions, integrin α5β1 increases RhoA activation from FAs or FBs, which increases their assembly and in addition promotes pulling on AJs (indicated by membrane deformation) by actin stress fiber formation and the recruitment of mechanosensitive proteins including vinculin, VASP, and zyxin. Integrin α5β1 also binds soluble ligands involved in barrier disruption, such as Ang-2, and elicits pro-inflammatory signaling through NF-κB, which increases FN deposition and further stimulates α5β1 ligation and signaling. This positive feedforward loop is further stimulated by a mechanosensitive complex consisting of PECAM-1, VEGFR2, and VE-cadherin and the mechanosensitive channel Piezo-1, which increase integrin activation in response to disturbed flow. α-act indicates α-actinin; AJ, adherens junctions; Ang-2, angiopoietin-2; β-pix, p21-activated protein kinase exchange factor beta; FAs, focal adhesions; FAJs, focal adherens junctions; FBs, fibrillar adhesions; IPP, ILK-PINCH-parvin complex; kind2, kindlin-2; NTM, Notch transmembrane domain; FN, fibronectin; LN, laminin; NF-κB, nuclear factor-κB; PECAM-1, platelet endothelial cell adhesion molecule-1; PTPRF, protein tyrosine phosphatase receptor type F; VASP, vasodilator-stimulated phosphoprotein; VEGFR2, vascular endothelial growth factor receptor-2; and vin, vinculin. Created with Biorender.com.

During inflammation, the expression of α5β1 and its main ligand fibronectin are strongly increased, and fibronectin binding to α5β1 elicits pro-inflammatory signaling in mouse arteries, in part through α5 cytoplasmic tail interactions with phosphodiesterase-4D5^146^ (Figure 6). The deposition of endogenous fibronectin by ECs, rather than circulating (liver-derived) plasma fibronectin, and the subsequent process of fibronectin fibrillogenesis by α5β1 drives pro-inflammatory gene expression and atherogenic progression in mice, in response to oxidized low-density lipoprotein. Inclusion of the EDA and EDB domains in cell-derived fibronectin is required for pro-inflammatory signaling.^147^ Thus, α5β1-fibronectin interactions create a positive feedback loop to maintain inflammation and promote leakage and atherosclerotic plaque formation, and blockade or haploinsufficiency of α5β1 reduces vascular leakage as well as formation of atherosclerotic lesions in mice.^148^

In contrast, enhanced vascular leakage and endothelial barrier disruption were observed in cultured HUVECs and human pulmonary artery endothelial cells treated with an αvβ3-blocking antibody and in β3-null mice in response to permeability-inducing agents including VEGF, while there was no effect in non-stimulated conditions.^145,149^ This is likely related to the observation that αvβ3 primarily supports Rac1, but not RhoA, activation. Rac1 activation reinforces cell–cell adhesion by promoting cell spreading and cortical actin polymerization at cell–cell contacts in HUVECs, pulmonary artery endothelial cells, and blood microvascular ECs.^143,145^ Integrin αvβ3 may also strengthen endothelial barrier function via mechanisms directly related to cell–matrix adhesion. HUVEC stimulation with TNF-α and interferon-γ reduced αvβ3-dependent adhesion to the ECM,^150^ while stimulation with thrombin activates signaling pathways disrupting αvβ3-dependent cell–matrix interaction.^151^ Furthermore, αvβ3 recruitment by junctional adhesion molecule-C in HUVECs destabilizes AJs,^151^ and its interaction with the Ang-2 receptor Tie2 in HUVECs induces αvβ3 internalization and degradation upon Ang-2 stimulation, contributing to endothelial destabilization and barrier dysfunction.^152^

Hemodynamic shear stress is an important determinant of the expression and activation of various integrins.^2,153^ Integrins have mechanosensitive properties, transduce mechanical signals to RhoA and Rac^2^ and act downstream of a mechanosensory complex consisting of PECAM-1, VE-cadherin, and VEGFR2.^154^ The type of shear stress (oscillatory versus laminar) and the composition of the ECM determine which integrin is activated and the downstream response. Laminar shear stress induces interaction of β1 integrins (among others α2β1 and α6β1) with laminins and collagens, resulting in cell alignment^155^ and increased cell adhesional strength.^33^ Laminins and collagens thus promote vascular homeostasis and suppress inflammatory signaling in artery endothelial cells.^156^ In contrast, oscillatory shear stress induces predominantly α5β1 activation and fibronectin deposition, resulting in the activation of YAP/TAZ (Yes-associated protein/transcriptional coactivator with a PDZ-binding domain)^157^ and NF-κB signaling^158^ in HUVECs and mice.^148,156^ Thus, oscillatory shear stress promotes inflammatory signaling through α5β1 and enhances its interaction with phosphodiesterase-4D5, resembling the mechanisms discussed above.^146^ Integrin activation through the PECAM-1/VEGFR2/VE-cadherin complex further requires the mechanosensitive ion channel Piezo1, as well as G_q_/G_11_ proteins^158^ (Figure 6). Indeed, the endothelial-specific deletion of Piezo1 or Gα_q_/Gα_11_ in mice reduced integrin activation, inflammatory signaling, and progression of atherosclerosis in atheroprone areas.^158^ Intriguingly, a mechanosensitive complex consisting of VE-cadherin, PTPRF, Notch and the Rac GEF Trio has been identified in AJs in HUVECs and human dermal microvascular ECs, and promotes local Rac activation and barrier function in response to flow^159,160^ (Figure 6).

In summary, multiple layers of crosstalk exist between cell–cell and cell–matrix adhesions, together regulating vessel development and stability in pathophysiological conditions. While β1 integrins stabilize cell–cell junctions, they can during inflammation also contribute to barrier disruption by (1) antagonizing αvβ3 function, (2) enhancing pro-inflammatory signaling through NF-kB, (3) assembling a fibronectin matrix, and (4) stimulating Rho-ROCK-dependent barrier disruption^143–147,154,158^ (Figure 6).

### Emerging Concepts of Endothelial Barrier Regulation by Cell–Matrix Adhesions

**Figure 7.**
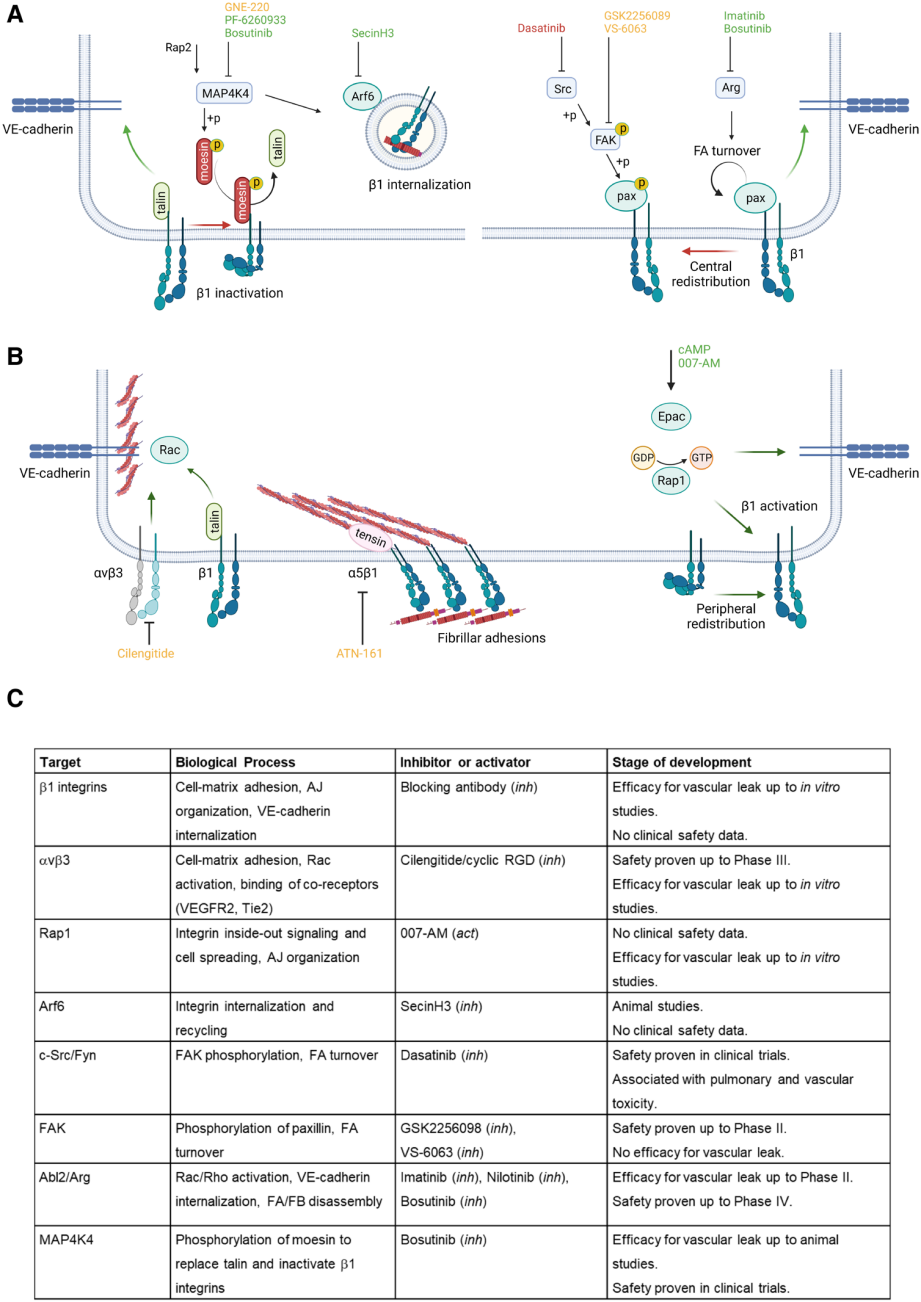
**Pharmacological intervention at cell–extracellular matrix (ECM) adhesions. A**, Targeting kinases and GTPases involved in turnover and spatial distribution of FAs. MAP4K4 is inhibited by several kinase inhibitors and phosphorylates moesin, which competes with talin for binding to the β1 integrin tail, thus favoring β1 integrin inactivation. MAP4K4 also activates Arf6 (inhibited by SecinH3), which drives endocytic recycling of β1 integrins, regulating their surface expression. c-Src, FAK, and the Abelson kinases are also inhibited by several tyrosine kinase inhibitors. c-Src and FAK regulate among others the phosphorylation of paxillin and FA turnover, involved in both barrier disruption and restoration. The Abl tyrosine kinase Arg increases FA disassembly and central redistribution of FAs. **B**, Integrin αvβ3 and peripheral, talin-bound β1 integrins enhance the integrity of VE-cadherin containing cell-cell junctions, protecting the endothelial barrier against barrier-disruptive agents. During barrier disruption, a talin-tensin switch parallels the formation of α5β1-containing FBs, which are located more centrally. While several compounds exist that target αvβ3 or α5β1, their potential role in barrier function remains to be established. The GTPase Rap1 can be activated by 007-acetoxymethyl and promotes integrin inside-out signaling, resulting in both integrin activation and cell spreading. Color coding for compounds and arrows: green indicates a barrier-protective effect, red indicates a barrier-disruptive effect, orange indicates compounds with unknown effect on endothelial barrier integrity. Created with Biorender.com. **C**, Summary of regulators of endothelial cell-matrix adhesion as targets for therapeutic intervention. *Act* indicates activator; AJ, adherens junction; Arg, Abl-related gene; cAMP, cyclic adenosine monophosphate; Epac, exchange protein activated by cAMP; FA, focal adhesion; FAK, focal adhesion kinase; FB, fibrillar adhesions; FN, fibronectin; *inh*, inhibitor; MAP4K4, Mitogen-Activated Protein Kinase Kinase Kinase Kinase; pax, paxillin; and VE-cadherin, vascular endothelial cadherin.

From the abovementioned studies, a number of consistent observations emerge. The subcellular localization of FAs correlates with endothelial barrier dynamics. While a large portion of FAs is distributed in the cell periphery during endothelial barrier recovery, FAs are predominantly located in the cell center during endothelial barrier disruption. This central redistribution is characterized by the formation of fibrillar adhesions, and a switch from talin- to tensin-containing adhesions (Figure 2C).^99,145^ At the same time that tensin-rich, centrally located fibrillar adhesions are formed, there is a temporal decrease in Rac1 activity, and concomitant disassembly of peripheral, talin-rich FAs.^145^ The disassembly of peripheral FAs is mediated by kinases like Arg^91^ and MAP4K4,^99,101^ and their depletion results in stabilization of peripheral FAs and improved barrier function (Figures 6 and 7). Peripheral cell–matrix adhesions and cell–cell adhesions preserve endothelial barrier integrity by together providing a tethering force to maintain cell spreading against internal tension caused by actomyosin contraction. Peripheral FAs also enhance cell adhesion strength to the ECM. This balance or reciprocity between cell–cell and cell–matrix interaction in maintaining spread cell shape may importantly determine the extent of endothelial barrier disruption upon inflammatory insults.

## Future Perspectives

### Translational Challenges

Insights into the molecular pathways underlying physiological cell–matrix interaction and inflammatory barrier disruption provide novel targets for intervention, with the ultimate aim to develop compounds that can be used in clinical setting to reverse vascular leak in syndromes like sepsis and ARDS. Developing drugs that target cell–matrix adhesion carries significant challenges. As discussed above, β1 integrins are ubiquitously expressed, being abundant not only in different vascular beds, but also in several nonendothelial cells. The clinical utility of compounds, however, strongly depends on their ability to target endothelial barrier integrity with relative specificity over other organs or tissues. These considerations seem to almost exclude a direct integrin-targeting approach, given its nonspecificity and the high probability of off-target effects. The largest translational challenge therefore seems the identification of integrin-regulating proteins that can be specifically targeted during endothelial hyperpermeability. As toxicity forms the major bottleneck for compounds entering clinical efficacy testing, there is a preference for evaluating drugs that are already used in clinical setting. The use of these de-risked compounds (drug repurposing) reduces risk of toxicity and at the same time lowers development costs and time. In this paragraph, we will discuss potential targets with their respective therapeutics, and their current status of development (Figure 7A through 7C).

### Targeting Integrin Function

The dynamic regulation of integrins during exposure of ECs to inflammatory insults may constitute a therapeutic target, although the complex and context-dependent functions of integrins may hamper systemic treatment. Blocking antibodies against β1 attenuated vascular leak and improved organ function in a mouse model of sepsis.^145^ However, the protective effects were obtained only at low concentrations, while higher concentrations interfered with AJ organization.^145^ This concentration-dependency may form an obstacle for clinical development of a therapeutic approach targeting β1 integrins, given the high variability in pharmacokinetics in critically ill patients. Reported effects of blocking αvβ3 in mice are more consistent, with disruption of the endothelial barrier^145^ and aggravation of lung injury and sepsis.^15^ In the absence of data on enhancing αvβ3 activity in animal models of vascular leak, little evidence supports the pursue of αvβ3 as therapeutic target. Moreover and in contrast to preclinical models, inhibition of αvβ3 with small molecules in clinical trials had little effect on tumor angiogenesis.^161^

Detrimental effects of targeting integrins may be circumvented by inhibiting integrin-regulating pathways, eg, the endosomal machinery or small GTPases. Examples include Arf6, which mediates integrin internalization and endothelial (in)stability, and can be inhibited by SecinH3, or the GTPase Rap1, which activates integrins via inside-out signaling, and can be activated by 007-AM.^162–164^ Both compounds have only been tested in preclinical models of endothelial barrier integrity, and have not been tested in clinical trials (Figure 7C).

### Targeting FA Turnover

FA turnover provides an alternative target for treatment of vascular leak, central regulators of which include small GTPases and protein kinases. Clinically available inhibitors of the RhoA effector ROCK have been discussed elsewhere.^3^ Although pharmacological activation of Rac1 and Cdc42 may be a potential strategy, the lack of specific activators hampers its further development.

Considering the role of kinases in FA turnover, the role of FAK has been characterized most extensively. Although clinical FAK inhibitors are available, the requirement of FAK for barrier restoration after initial gap formation precludes the use of FAK inhibitors in clinical conditions of vascular leak. Alternatively, the tyrosine kinase Arg was identified as regulator of endothelial FA turnover^91^ following the observation that the clinically available Abl kinase inhibitor imatinib protects the endothelial barrier under inflammatory conditions.^88^ Imatinib enhanced integrin-mediated adhesion and barrier function of HUVECs and human pulmonary microvascular ECs, and restricted vascular leakage and pulmonary edema in mouse models of sepsis and ARDS.^88,90,165^ Imatinib has been used for 20 years in the treatment of chronic myeloid leukemia with a favorable safety profile at dosage up to 800 mg/day. In 2 clinical cases, imatinib reduced pulmonary vascular leak at 200–400 mg,^131,166^ without apparent side-effects. Recently, imatinib was evaluated in a randomized, clinical trial in 385 COVID-19 patients requiring supplemental oxygen (COUNTER COVID study). Patients were treated with imatinib for 10 days after hospital admission. Although the primary endpoint (time to liberation from supplemental oxygen) was not met, signals of benefit were observed in patients with severe course of disease (COVID ARDS), including a reduction in mortality and a reduction in the time on the mechanical ventilator.^167,168^ Currently, the effect of imatinib is being evaluated in the SOLIDARITY and REMAP-CAP platform studies, and in the INVENT COVID clinical trial.^169^ Second-generation Abl kinase inhibitors like nilotinib have shown similar protective effects on the endothelial barrier.^170^ Although this is indicative for a class effect of Abl kinase inhibitors, the inhibition of multiple targets by these inhibitors may result in a wide array of endothelial effects.^171^ This is further enhanced by the varying half maximal inhibitory concentration of different kinase inhibitors, leading to inhibitory profiles that strongly depend on the inhibitor concentration.

Another regulator of FA turnover is MAP4K4, which was effectively inhibited by the pharmacological inhibitors PF-6260933^101^ and GNE-220^99^ in HUVECs and in mice (Figure 7C). While these inhibitors are not clinically available before safety testing in early clinical trials, the tyrosine kinase bosutinib was identified as an alternative MAP4K4-targeting drug, and is already in clinical use.^101^ Bosutinib inhibits both Abl2 and MAP4K4, and in line with MAP4K4 and Abl2 depletion, treatment of HUVECs with bosutinib strongly reduced FA disassembly both under basal conditions and during thrombin stimulation. The barrier-protective effect of bosutinib was partly abrogated by co-treatment with integrin-blocking peptides,^101^ again underlining the role of cell–matrix adhesions in endothelial barrier regulation. In mouse and rat models of acute lung injury and trauma-induced vascular leak, bosutinib reduced pulmonary vascular leak, organ function, and systemic hypotension.^101,172^

Finally, the observation that many biologically relevant inhibitors fail to reach the clinical stage of development challenges the classical bottom-up approach for drug development, that is, preclinical identification of a target, followed by development of an inhibitor with subsequent clinical testing. As stated above, repurposing of clinically available drugs or shelve compounds that have passed safety testing may be a time- and cost-effective approach to develop novel therapeutic strategies. With the availability of large databases (both compound libraries and disease expression profiles), the serendipity that was historically associated with drug repurposing has been largely replaced by forced serendipity by computational approaches. Disease expression profiles (transcriptomics, proteomics, or phosphoproteomics) can be matched to drug expression profiles (eg, transcription profiles after exposure to a compound). Signature matching is the analysis in which disease signatures are compared with a large database of drug signatures in order to identify compounds that can reverse the disease signature. The other way around, repurposed drugs may reveal new targets and generate novel hypotheses on disease mechanisms. The latter is exemplified by a recent study in human brain microvascular ECs and HUVECs, which used polypharmacology of clinically available kinase inhibitors to dissect endothelial kinases modulating endothelial barrier integrity. In a kinase regression approach, the endothelial barrier-modulating properties of 28 kinase inhibitors with divergent barrier responses were analyzed. Targeted deconvolution revealed 50 kinases involved in endothelial barrier regulation, 30 of which had not been linked to endothelial barrier regulation before.^173^

## Conclusions

Endothelial barrier disruption is frequently observed during angiogenesis and complicates inflammation, leading to vascular leak as driver of morbidity and mortality in several diseases, including sepsis and ARDS. While cell–cell junction integrity is an established determinant of endothelial barrier stability, cell–matrix interaction may be of additional/equal importance for maintenance of the endothelial barrier. The involvement of integrins in barrier regulation has gained new interest in recent years, and a number of topics have emerged in this review, which deserve attention in the near future to better understand the contribution of cell–matrix interaction to endothelial barrier function and the cross-talk between cell–matrix adhesions and cell–cell junctions. First, in contrast to α5β1 and αvβ3, the relevance of several other integrin heterodimers in endothelial barrier regulation is less clear. Second, the exact role of some integrin heterodimers in endothelial barrier regulation is strongly context-dependent, while the determinants of this context remain less known but include subcellular distribution, intracellular binding partners, and co-receptors. Third, as direct integrin targeting as therapeutic strategy may carry a high risk of toxicity, the machinery and signaling pathways involved in integrin function and dynamics may form a more suitable target. Altogether, unraveling the mechanisms regulating cell–matrix adhesion has yielded and will yield novel targets for treatment of impaired endothelial integrity, in which regulatory proteins like small GTPases and kinases seem most promising as candidates for treatment.

## Article Information

### Acknowledgments

We sincerely apologize to the many colleagues whose work could not be cited due to space constraints. We are grateful to Arnoud Sonnenberg and Victor W.M. van Hinsbergh for critical reading.

### Sources of Funding

Work in our laboratories is supported by the Landsteiner Foundation for Blood Transfusion Research (CM; grant no. 2019), NWO ZonMW (JA; VENI grant no. 09150161910155), and the Dutch Lung Fund (JA; grant no. 11.1.20.025).

### Disclosure

None.
